# Advances in Regenerative Stem Cell Therapy in Androgenic Alopecia and Hair Loss: Wnt Pathway, Growth-Factor, and Mesenchymal Stem Cell Signaling Impact Analysis on Cell Growth and Hair Follicle Development

**DOI:** 10.3390/cells8050466

**Published:** 2019-05-16

**Authors:** Pietro Gentile, Simone Garcovich

**Affiliations:** 1 Surgical Science Department, Plastic and Reconstructive Surgery Unit, University of “Tor Vergata”, 00133 Rome, Italy; 2 Institute of Dermatology, F. Policlinico Gemelli IRCSS, Università Cattolica del Sacro Cuore, 00168 Rome, Italy; simgarko@yahoo.it

**Keywords:** stem cell therapy, stem cell hair loss, human follicle stem cells, platelet-rich plasma, hair loss, hair regrowth, PRP hair, stem cells hair

## Abstract

The use of stem cells has been reported to improve hair regrowth in several therapeutic strategies, including reversing the pathological mechanisms, that contribute to hair loss, regeneration of hair follicles, or creating hair using the tissue-engineering approach. Although various promising stem cell approaches are progressing via pre-clinical models to clinical trials, intraoperative stem cell treatments with a one-step procedure offer a quicker result by incorporating an autologous cell source without manipulation, which may be injected by surgeons through a well-established clinical practice. Many authors have concentrated on adipose-derived stromal vascular cells due to their ability to separate into numerous cell genealogies, platelet-rich plasma for its ability to enhance cell multiplication and neo-angiogenesis, as well as human follicle mesenchymal stem cells. In this paper, the significant improvements in intraoperative stem cell approaches, from in vivo models to clinical investigations, are reviewed. The potential regenerative instruments and functions of various cell populaces in the hair regrowth process are discussed. The addition of Wnt signaling in dermal papilla cells is considered a key factor in stimulating hair growth. Mesenchymal stem cell-derived signaling and growth factors obtained by platelets influence hair growth through cellular proliferation to prolong the anagen phase (FGF-7), induce cell growth (ERK activation), stimulate hair follicle development (β-catenin), and suppress apoptotic cues (Bcl-2 release and Akt activation).

## 1. Introduction

Hair tissue engineering and stem cell therapy are new approaches to treating hair loss (HL). Methods using exogenous cell sources or progenitor cells (PCs) are being tested in cell treatment clinical trials. These trials incorporate cells obtained from allogeneic and autologous sources. Specifically, intra-surgical cell treatments that incorporate autologous cell-based treatments with a one-step approach (cell harvesting, minimal manipulation, and immediate injection) into a single technique offer tremendous potential; a few methodologies have achieved clinical application. The intra-surgical cell treatment process involves tissue collection and preparation to obtain the desired cell product, followed by careful evaluation using the clinical application, and then cell conveyance. Intra-surgical cell treatment benefits from the availability and safety of using the patient’s own cells, which do not trigger an adverse reaction, as well as from the numerous important cell types that can be harvested using minimally invasive strategies [1]. 

This treatment bypasses a significant number of restrictions associated with exogenous cell treatment by avoiding in vitro cell control and expensive cell extension, the requirement for good manufacturing practice (GMP) facilities, the need to procure a work force for cell culture preparation, the potential for pollution, and a second method (at an alternate time point) to collect the cells. It might be helpful to maintain a strategic distance from the cell culture to restrict phenotype changes that may occur when cells are expelled from their local microenvironment for an all-encompassing time period [1]. 

Additionally, the techniques are entirely performed inside the surgical room (in absence of culture growth), which may lessen the hold-up time for the medical procedure. The U.S. Food and Drug Administration (FDA), the European Medicines Agency (EMA), and other administrative specialists consider grown-up cell products as biological products that can be partitioned into two classes: minimally manipulated biological products (obtained through centrifugation, filtration, and isolation without cell expansion) and manipulated biological products (obtained through culture-expanded stem cells). Certain intraoperative cell approaches fit the minimally-manipulated biological product category in which broad clinical trials are not required, consequently speeding up potential interpretation to facilities.

## 2. Hair Loss and Androgenic Alopecia: Bio-Molecular Pathway Disorder

HL is determined by an assortment of factors: inherited (trichodystrophy and androgenetic alopecia), accompanying comorbidity conditions, hormonal clutters (thyroid organ disease, insulin resistance), immune system (patchy alopecia and lupus erythematous), nutritional scatters, environmental elements (drugs, ultraviolet (UV) radiation), mental disorders (stress and trichotillomania), and aging. Harming factors influence the hair cycle (HC) and reduce stem cell activity and hair follicle recovery capacity [2]. 

A clinical need exists for the advancement of biotechnology to enhance hair growth to address the HL issue, specifically, in cases of androgenic alopecia (AGA). 

AGA is a dynamic and incessant HL issue affecting 80% of white males and 40% of white females below the age of 70, in which lymphocytes and mast cells have been identified around the miniaturizing follicle in the stem-cell-rich knot zone [3,4,5,6]. The scaling down of the follicles (i.e., miniaturization) is characterized by a depletion of the anagen stage, with an enhancement in the measure of resting hair follicles (HF), telogen, and the presence of infinitesimal hairs on a bald scalp [5,6,7]. 

Current drugs available to treat AGA include medications such as Finasteride^®^; topical moisturizers such as Minoxidil^®^, and surgical procedures such as hair transplantation [4]. In HL scalps, the hair follicle stem cell numbers remain unaltered; however, the quantity of the more effectively multiplying PCs significantly decreases [7]. 

Along these lines, the aim of hair tissue engineering is developing new autologous advancements to induce hair regrowth by in vitro and ex vivo cultures or by in vivo recovery and bio-stimulation. Autologous stem cells (SCs) have been of particular interest for application in hair regrowth. Some early endeavors in the field concentrated on disengaging essential cells from a biopsy of the scalp tissue and maturing the cells ex vivo for a resulting injection into the patient. 

Alopecia includes modifications to two sorts of hair SCs, represented by the hair follicle stem cells (HFSCs) and the dermal papilla cells (DPCs) [8,9]. 

HFSCs and DPCs guarantee conditions for appropriate hair recovery and regeneration [9]. In scarring alopecia (lupus erythematous, lichen planus), provocative cell invasion around the bulge results in an irreversible loss of HFSCs. Despite the PCs being harmed, HFSCs are saved in patchy and AGA, and this is the reason why this kind of HL can be reversible [9]. 

Bulge SCs have been progressively portrayed, particularly in murine HFs, thereby promoting their recognition, although no widespread marker has been found for them. An example is cytokeratin 15 (CK15), which explains why CK15+/integrin α6+ or CD34+/integrin α6+ cells have been distinguished as bulge cells [10]. 

Research on murine HFs has shown the expression of CK19 [11,12] and various transcriptional factors, that is, Gli1, Sox9, LHX2, Hopx, Tcf3, and Nfatc1, [11,13]. The expression of specific markers relies on the HC stage and on the exact area of the cells inside the bulge [11,14]. Lgr5, a receptor engaged in the Wnt signaling pathway, has been distinguished as a genuine marker of HFSCs [13]. 

The SCs of the upper and lower parts of the bulge in telogen HFs influence the expression of CD34 and part of the lower portion of Lgr5. Cells that are involved in the growth of another anagen hair express Lgr5 and not CD34 [15]. 

Cells of the upper piece of the bulge present a higher expression of Nfatc1, which is related to a condition of rest [6]. Expression of Lgr6 [14,16] and Lrig1 [14,17] has been observed inside the isthmus. The PCs of the germinal matrix are obtained from the SCs of the bulge, but unlike them, the PCs display a high grade of P-cadherin [11,18]. 

Human HFSCs (H-HFSCs) are less known than murine HFSCs (M-HFSCs). It appears that specific markers are normal in both human and mouse HFSCs: CD34 [19,20], K15 [8,19], K19 [19,21], and CD200 [8,19,20]. The presence of different markers (i.e., Sox9 and LHX2) requires further examination [22]. Markers found only in H-HFSCs are PHLDA1 [19,23] and EpCAM/Ber-EP4, which is considered a valuable marker of the telogen optional hair germ [18,19]. Dermal papilla (DP) cells present distinctive markers, including the cells from hair follicle cells (HFs) and dermal fibroblasts [24]. Alkaline phosphatase (ALP) is critical for both human and murine HFs, and it is the most explicit of the markers [21,22,24]; wherein its high action is considered a marker of DP cell separation [24,25]. Additionally, α-SMA [24,25], laminin, ad fibronectin [24], and CD133 [24,26] expression have been observed in DPCs. 

Marker expression modify in disease states. The immunoreactivity of CK15 is diminished in individuals with patchy alopecia, and is also identified in AGA [10]. HF of the scalp’s front part displays a shortage of CD34 in AGA, whereas its appearance is conserved in HF of the occipital locale [10]. In patchy alopecia, CD200, another marker of matrix cells, is expressed ineffectively, which might be an indication of a reduction in the immune benefit contributing to pathogenesis (i.e., response of auto reactive lymphocytes) [10,27]. 

SCs in the bulge remain in the resting stage for the vast majority of their lives, yet they can be actuated by relying upon the HC stage. Several theories with respect to the course and control of the HC have been proposed amid research in mouse models [12]. From the HC in mice, in the anagen stage, SCs in the bulge are separated multiple times and remain inside the niche, whereas cells of the germinal matrix divide strongly and produce the maturing hair shaft. During the catagen stage, cells from the germinal matrix experience apoptosis, while SCs of the bulge move out of it to the outer HF and, in this manner, toward the finish of the catagen stage, where they shape another bulge around the hair stem and another germinal matrix under the bulge. SCs in the bulge remain in a condition of rest during the telogen stage, and between the telogen and anagen stages, where they self-recover or move, creating a pool of germinal framework cells that multiply to shape the hair matrix [28]. 

The priority is on derivative cells in the bulge, which are the alleged SCs progenitor cells of the germinal matrix in the outflow of genes that influence stem cell activation, which have priority in expansion during the recovery cycle, even before the cells in the bulge [11,29,30]. 

The interpretation of the HC into the human HC has a few constraints due to the distinctive lengths of anagen [31,32], asynchrony of the human cycle [31,33], or the alternate response to the impact of hormonal variables [31,34]. Different papers reported the results of human scalp skin xenografted onto immune-compromised mice to set up the HC course in vivo in people [31,35]. 

The action of SCs in the bulge is controlled by its microenvironment (i.e., a supposed niche). This microenvironment incorporates the daughter cells of the SCs in the bulge, which enact their self-recovery ahead of schedule and in the late anagen stages [36]. SCs are fundamentally influenced by the mesenchymal cells (MCs) of the DP, which are in close contact with the cells of the germinal matrix that are isolated by the basal membrane [14]. They appear to be of vital significance in the activation of hair growth and in signal transmission during recovery [11,30]. 

Investigations have demonstrated that hair recovery is impossible after laser treatment, on the grounds that the HF cycle stops at the telogen stage without advancing to the anagen stage [14,28,30,37]. Infusions of exosomes obtained from DPCs to HFs have been found to accelerate the passage of anagen and catagen delay by means of the β-catenin and Shh pathways [38]. HFSCs are additionally influenced by fibroblasts in the reticular and papillary layers of the dermis, in addition to the subcutaneous tissue [14]. Inside the niche, there are melanocyte SCs that are in charge of the formation of mature melanocytes that confer color to maturing hair. The survival and growth of MSCs relies on signs transmitted by the HF epithelial cells (ECs), for instance, the TGF-β or the Wnt pathway [14,36]. The extracellular matrix is another part of the microenvironment. It specifically influences the SCs through the arrangement of the basal layer, in which undifferentiated cells are in contact and regulated, for instance, by integrins [11,28].

## 3. SCs Use in HF Regeneration

HFs are immunologically-favored spots, similar to the cerebrum, eyes, and gonads, and they are influenced by the neuroendocrine-immune system [26]. In physiological conditions, this is influenced by: (1) Low expression or non-appearance of the principle MHC I antigens, (2) the presence of malfunctioning Langerhans cells, and (3) local expression of immunosuppressive substances (TGF-β1 and α-melanocytes MSH) [26,39]. Inferred from this is that HFs can be effortlessly used in transplantation. 

Multipotent SCs can re-generate HFs with sebaceous organs in the skin. Given current information, SCs can be used to regenerate hairs in a few therapeutic methodologies such as: (1) Reversing the pathological mechanisms that determine HL (particularly in AGA), (2) regenerating mature HFs from their parts (cells in the bulge can regenerate an entire hair), and (3) neogenesis of HFs from a SC-culture with separated cells or tissue designing [40,41,42].

## 4. FDA and European Rules Regarding Use of Adipose Derived-Stromal Vascular Cells (AD-SVFs) and Human Follicle Mesenchymal Stem Cells (HF-MSCs) 

The U.S. Food and Drug Administration (FDA) and the European Medicines Agency (EMA) consider grown-up cell products as biological products that are isolated into two classes: minimally manipulated biological products and manipulated biological products. Regulation number 1394/2007 of the European Parliament for cutting-edge treatments defines “bioprocess engineering products,” which excludes products that contain or are made solely of cells and non-vital human or animal tissues that do not have pharmacological, immunologic, or metabolic activity. Included amongst the advanced therapy pharmaceutical products are ones used for gene and somatic cell treatment (Directive 2001/83/European Parliament, Annex I). Cells and tissues are to be viewed as results of the bioprocess engineering products in the event that they experience “extensive manipulation”. This rule contrasts between extensive and minimal manipulation. Manipulations that are not considered as bioprocess engineering include the following: cutting, granulating, forming, purification, centrifugation, absorbing anti-toxins or antimicrobial arrangements, cleansing, lighting, partition, fixation or decontamination, filtration, lyophilization, solidifying cryopreservation, and nitrification. The definition of medicines for advanced therapy excludes non-repetitive preparations completed under the supervision of a doctor running an individual remedy for a product explicitly intended for that specific patient, without obviously disregarding the important standards that identify with quality and security.

Further to the execution of Article 17 of Regulation (EC) No 1394/2007 (the Advanced Therapy Medicinal Products (ATMPs) Regulation), applicants are required to approach the Committee for Advanced Therapies (CAT) with a logical proposal for the arrangement of ATMPs. The committee is in charge of surveying the quality, well-being, and viability of cutting-edge treatment medications, including medications delegated as quality treatment, substantial cell treatment, or tissue-built products. CAT is supported by the ATMP regulation, which empowers the EMA in a joint effort with the European Commission to decide if a given product meets the logical criteria that characterize ATMPs. The ATMP grouping technique was introduced with the goal of addressing inquiries into situations where the arrangement of a product dependent on genes, cells, or tissues is not clear. The CAT issues logical proposals to determine if the product falls within the definition of an ATMP in the European Union. The ATMP Regulation and Directive 2001/83/EC Annex I Part IV [30] provide exact legal definitions of ATMPs. 

The ATMP characterization depends on an assessment of whether a given product satisfies one of the characteristics of gene therapy medicinal products (GTMP), somatic cell therapy medicinal products (sCTMPs), or tissue engineered products (TEPs), and whether that product satisfies the definition of a consolidated ATMP. It is additionally recognized that, because of the complex nature of these restorative products, the constrained information bundle at the beginning period of the product improvement, as well as the rapid growth of science and innovation, may result in inquiries off the fringe.

### 4.1. EMA/CAT Recommendations on Minimal Manipulation

According to the reflection paper on the portrayal of front-line treatment therapeutic products, EMA/CAT/600280/2010 Rev 1, June 20, 2014, by the Committee for Advanced Therapies (CAT), Line 10, “a similar basic capacity for a cell populace implies that the cells, when expelled from their unique condition in the human body are used to maintain the original capacity in a similar anatomical or histological condition.” The authors presumed autologous application in a one-stage medical procedure, minimal manipulation, omofunctional use “used for an indistinguishable fundamental capacity in the beneficiary as in the donor”, and manipulation with devices in aseptic conditions would be conditions that do not require good manufacturing practice (GMP) rules for preparation, good clinical practices (GCP) for the clinical application, or ethical committee underwriting.

### 4.2. Italian Rules Regarding Platelet-Rich Plasma Use

Platelet-rich plasma (PRP) preparation must be performed in Italy respecting the Decree of the Blood, November 2, 2015, dispositions related to quality, and safety parameters for blood and emocomponents.

## 5. Cell and Growth Factor Sources

Adult stem cells and PCs can be intra-surgically collected from a few tissues, including fat tissue, scalp tissue, bone marrow (BM), and peripheral blood. Previously, BM was viewed as a widely recognized source for these cells, since it was effortlessly and quickly obtained and because several devices were available for gathering BM. BM contains hematopoietic cells and MCs, as well as different cells types that may be involved in advancing tissue regeneration and hair regrowth. One of the primary constraints of intra-surgical SCs treatment approaches was the restricted amount of reaped source material and the low yield of cell detachment conventions, which yielded SCs at a frequency per mono-nucleated cell of one in every 10,000 cells to one in every 100,000 cells [43,44,45,46]. To overcome these difficulties and to avoid the highly invasive procedure of BM harvesting that causes pain at the donor area, additional elective sources from which to separate autologous SCs ought to be considered. 

Fat tissue is an interesting source of cells that have multi-lineage separation potential. This tissue tends to be harvested using a less invasive technique, represented by liposuction and in larger amounts than in BM [47]. In fact, fat tissue harvest is much easier and less painful for the patient compared with BM gathering, in which it is necessary to use a trocar to drill the iliac crest [47]. A few investigations have reported different examinations and analyses between SCs obtained from various sources [47,48,49,50]. Peng et al. [47] included an immunophenotypic investigation of SCs separated from rodent BM and fat tissue, and uncovered a noteworthy level of CD44+, CD73+, and CD90+ cells in fat tissue. Increased multiplication potential in cells detached from fat tissue was proposed, citing a higher and steadier growth rate all through 10 generations, a lower populace-doubling time, the highest proportion of cell populace in the S stage, and higher telomerase activity. These outcomes were confirmed by other examinations that emphasized the evaluation of various grownup, undifferentiated cell sources [48,50]. Fat tissue must be viewed as a real alternative to BM for intra-surgical use based on SCs wealth and expansion potential. A few patients may have constrained or minimal fat tissue for autologous settings, given the high frequency of adipose tissue-derived MSCs (their occurrence is 100 to 300 times higher than in bone marrow, and the number of SCs that can be counted per unit volume of fat harvested is approximately 10-fold greater than that from BM), small fat tissue repositories may still be adequate for SCs separation [48,51,52,53]. When gathering fat tissue for intraoperative SCs treatment, the tissue reaping site and the surgery affect the yield of undifferentiated cells. For example, fat tissue harvested from the abdominal region through resection or liposuction yields more SCs in contrast with ultrasound-assisted liposuction and with the fat tissue collected from the hip/thigh district [52].

For scalp tissue, in the preliminary examination performed by Cole J.P. et al. [53], they developed another system to separate human adult SCs with minimal manipulation depending on the centrifugation of fragments of human HFs without extension or culture. Specifically, the authors reported, for the first time, the results obtained in a hair regrowth study using a therapeutic device called Rigeneracons^®^ (CE confirmed Class I, Human Brain Wave, Turin, Italy) that was used to obtain autologous micrografts containing HF-MSCs from centrifugation of a punch biopsy of the scalp, easily accessible for use in patients affected by AGA. The micrograft units were obtained by the disaggregation of a 2 mm punch biopsy by selection of a cell populace with a diameter of 50 microns. High cell viability was reported [53]. Noteworthy limitations include the challenge in growing cells to adequate numbers for human use, the need to conduct this expansion in good manufacturing practices (GMP) research centers, and the viability of the extended cells [54].

Therefore, the clinical use of HF-MSCs to enhance hair regrowth has not been satisfactorily considered. In other works [40,53], authors cited the amount of CD44+ cells (hair follicle-determined mesenchymal SCs) from the DP, and the level of CD200+ cells (hair follicle epithelial-SCs) from the bulge, obtained by means of the customized centrifugation of 11 punch tests [40,53]. The authors reported the microscopic evaluation of punch biopsy samples, performed using cytospin, immunocytochemistry, and the histological examination achieved by hematoxylin and eosin staining and clinical appraisal, where they discussed improvements to the current systems available for the recovery and regeneration of hair follicles. The authors emphasized permitting neo-genesis of HFs in adult individuals using isolated cells and biotechnologies [53]. 

The use of growth factors in autologous platelets was determined to provide substantial help in hair tissue regeneration owing to the platelets’ ability to advance neo-angiogenesis, cell expansion, and separation [55,56,57]. Platelet-rich plasma (PRP) contains no less than six fundamental growth factors, including the fibroblast growth factor (b-FGF), platelet-derived growth factor (PDGF), vascular endothelial growth factor (VEGF), epidermal growth factor (EGF), transforming growth factor-β (TGF-β), and insulin-like growth factor-1 (IGF-1) discharged after platelet actuation [58]. Every one of these significant growth factors is engaged in an explicit bio-molecular activity.

### Bio-Molecular Pathway of Stem Cells and Growth Factors That Improve Hair Regrowth 

Hair regrowth was regulated prevalently by the Wnt biomolecular pathway and ERK activation in which SCs and growth factors are involved as reported following:

- Hepatocyte growth factor (HGF) and HGF activator (discharged by DPC) enhance the proliferation of follicular ECs;
- EGF improves the activity and growth of follicle outer-root sheath cells by activation of Wnt/β-catenin flagging;
- b-FGF improves the advancement of hair follicles;
- Interleukein-6 (IL-6) is involved in WIHN through STAT3 enactment;
- VEGF improves peri-follicular angiogenesis;
- TGF-β stimulates the signaling pathways that manage the HC;
- IGF-1 improves the migration, survival, and proliferation of HF cells;
- IGFBP-1 to -6 manages the IGF-1 effect and its connection with extracellular matrix proteins at the HF level;
- BMP maintains the DPC phenotype (fundamental for stimulation of HFSCs);
- BMPR1a maintains the proper identity of the DPCs (basic for explicit DPC work);
- M-CSF is involved in wound-induced hair growth;
- M-CSFR is involved in wound-induced hair growth;
- PDGF and PDGFR-β/-α64 up-regulate the genes associated with HF separation, induction, and control of anagen. PDGF and its receptors are fundamental for follicular improvement;
- Wnt3a is involved in HF advancement through β-catenin flagging;
- PGE2 stimulates anagen in HF;
- PGF2α and analogs enhance change from telogen to anagen;
- BIO (GSK-3 inhibitor);
- PGE2 or hindrance of PGD2 or PGD2 receptor D2/GPR4477 enhances follicle regeneration; and
- Iron and l-lysine95 (still under examination).

## 6. Clinical Intra-Surgical Applications of AD-SVFs in Hair Loss and Androgenic Alopecia

Another field involves the possibility of using fat and stromal vascular fraction cells (SVFs) for hair regrowth. SVFs are a heterogeneous gathering of non-cultured cells that can be constantly isolated from fat using minimal manipulation, using centrifugation, filtration, and the purification of fat tissue (proposed by EMA-CAT), or using enzymatic digestion (not proposed in the EMA-CAT suggestions). These cells work through paracrine frameworks to improve adipocyte suitability. Festa et al. [59] reported that adipocyte ancestry cells support stem cell function and help to drive the hair growth cycle. This follicular regenerative methodology is incipient and raises the probability that the HC in male and female HL can be driven or restored using autologous fat enhanced with SVF. Perez-Meza et al. [60] reported the safety and tolerability of these fat graft procedures in patients affected by inherited alopecia treated with sub-cutaneous scalp injections of fat tissue. An augmentation of 31.0 hairs/cm^2^ was recorded in patients receiving a treatment of fat enriched with SVFs, where one subject who received fat alone (without SVFs addition) revealed a mean improvement of 14.0 hairs/cm^2^, suggesting that fat grafts could be a treatment method for early HL, where the fat enhanced with SVFs may improve this response [60]. These studies suggest that scalp SVF-enhanced fat grafts could be a promising elective approach to treating HL in individuals. Fukuoka et al. [61] investigated the effects of fat-derived stem cell-conditioned medium injection in a gathering of 22 patients affected by AGA. Patients received treatment every three to five weeks for a total of six sessions. The mean augmentation in the hair number (hairs/0.65 cm^2^) was 29 ± 4.1 in men and 15.6 ± 4.2 in women. No significative difference was observed between men and women. 

An examination of the studies suggests that HF neo-genesis using human ECs and dermal cells is an extremely cumbersome process that requires novel culture conditions, somehow replicating the conventional or embryonic skin conditions and the use of embryonic or neonatal cells. There are no tissue regeneration protocols used in hair transplants involving the use of AD-SVFs. Zanzoterra et al. [62] analyzed the capacities of cell solution in the Rigenera^®^ (Human Brain Wave, Turin, Italy) framework, which was obtained by the mechanical fracture of subcutaneous and fat tissue from the occipital region. The cell solution was injected into the hair transplant region, thereby expanding the measure of growth factors. Microscopic damage was seen to resolve more rapidly and the transplanted hair matured constantly, even two months after the strategy, wherein the telogen stage was abbreviated [62]. 

AD-SVFs-conditioned medium (AD-SVFs-CM) was used for patients with HL in an examination by Fukuoka and Suga [63]. A commercial product containing a protein arrangement with AD-SVFs was used (AAPE, Prostemics, Seoul, Korea), which had different growth factors (HGF, FGF-I, granulocyte colony-stimulating factor (G-CSF), granulocyte macrophage-colony-stimulating factor (GM-CSF), IL-6, VEGF, and TGF). The treatment (0.02 mL/cm^2^ of the arrangement) was performed by injecting the suspension intradermal every three to five weeks (for four to six sessions), and hair growth was checked using trichograms. A real enhancement in hair thickness was accomplished in patients of both sexes [63]. Shin et al. [64] used AD-SVFs and the conditioned media of AD-SVFs (AD-SVFs-CM) in an observational examination of 27 women with female pattern hair loss (FPHL). The use of AD-SVFs-CM demonstrated efficacy in treating FPHL following 12 weeks of treatment, citing improvements in hair thickness and hair density without serious side effects [64]. Won et al. [65] also demonstrated that the use of AD-SVFs-CM upgraded the multiplication of cultured human DPCs by up to 130% [65]. 

Different examinations confirmed that enhancing fat tissue with SVFs supports adipocyte viability and yields better outcomes in a hair transplant treatment when they are applied in grafts [66,67].

### 6.1. AD-SVFs Regenerative Mechanisms 

Recognizing the components that intervene in the impacts of intraoperative fat stem cell treatments is a problematic task. Current information that provides significant determinations or conclusions is lacking. This is not surprising as most of the fat SCs treatment evaluations focus on the appraisal of security and feasibility, and generally disregard the examinations aimed at understanding the mechanisms responsible for the results. Potential mechanisms involved are: (1) SCs or PCs inside the transplant renew the PCs in the host; (2) cells in the transplant differentiate and deliver new tissue; (3) transplanted cells emit trophic, angiogenic, or immunomodulatory factors that provide signals to nearby endogenous cells or distant cells, in the first case by means of paracrine signals, or in the second case (distant cells) by means of the endocrine mechanism (which may result in the assembly and homing of distant host cells); and (4) it is additionally conceivable that transplanted cells may combine with host cells (in a procedure known as cell combination). 

The explanation of the mechanisms of the functional improvements is convoluted by the heterogeneous aspect of a significant number of the intra-surgically-injected cell populaces, where certain cell types may play a predominant role or various cell types may cooperate synergistically. In particular, as numerous intra-surgical cell treatment approaches use heterogeneous cell populaces that may incorporate stem or PCs, it is conceivable that positive results may not be mediated by the stem or PCs. Likewise, as cells are detached and handled inside the operating room, there is likely extensive variation in how the cells are acquired, prepared, and mixed (regardless of endeavors to institutionalize methods with all-around structured packs and equipment). This variance may affect the method of activity and results. Intraoperative cell treatments include disconnection of autologous cells from their source, resulting in noteworthy contrasts in the relative cell numbers and phenotypes, which may be derived from the different age, sex, race, weight list, family/hereditary genes, diet, and condition of the donors. The mechanism also relies on the type of infusion (e.g., manual versus mechanical and controlled) and isolation technique (e.g., enzymatic versus centrifugation and filtration). Along these lines, numerous inquiries remain unanswered, and new strategies and in vivo studies will be required to explain how transplanted cells mediate restorative reactions [68]. Almost certainly, the systems and mechanisms used to control the destiny and capacity of cells following transplantation (e.g., by designing exogenous cells inside the operating room) [69] will provide a line to follow to amplify the restorative impact. These techniques should help lessen the variability of the treatment response.

### 6.2. Adipose Tissue, Adipocyte, and AD-SVFs Potential Roles in Hair Loss

HFSCs are additionally influenced by the overall macro-environment encompassing the HF and fat tissue. Adipose tissue appears to experience comparative changes compared to the HF. The thickness of the fat tissue increases during the anagen stage and the adipocytes multiply greatly [11,70]. Adipocytes emit BMP2 during the late catagen stage and early telogen stage, which supports the resting states in the niche, whereas emission of BMP2 is lessened toward the finish of the telogen stage, which bolsters the activation of HF-MSCs [11,70,71]. 

Correspondence between fat tissue and the epithelium keeps running in the same line. Transformations hindering the HC have been found to restrain adipogenesis, which suggests that epithelium cells send signals actuating the expansion of the adipocytes [28,70]. The HFs absorb supplements from the micro-vascular network, which is changed amid the HC; angiogenesis expands during the anagen stage [28,71]. Bulge cells and the matrix may likely invigorate angiogenesis [28]. Delayed activation of angiogenesis, which is associated with impaired angiogenesis, has been observed in mice [14,71]. It has been recommended that SCs prefer low-oxygen conditions, where they emit markers of hypoxia [28,72]. 

The vascular system, particularly that encompassing the isthmus containing venous vessels, may participate in maintaining the low-oxygen conditions in the areas surrounding the SCs environment [28]. Although the impact of the immune reaction has not been adequately illustrated, it is critical that the job of maintaining the immune benefits of HF related to the diminished expression of MHC I molecules and to the improved discharge of immune-suppressors ought to be maintained during the anagen stage [28,35]. The loss of this benefit and a safe assault on cells of the matrix and the bulge are related to alopecia [28,39]. Dermal cells γδT are known to regulate post-traumatic regeneration of HF by discharging FGF9 [14,73]. Therefore, macrophages increase the dimension of Wnt7b and Wnt10a ligands during the telogen stage after experiencing apoptosis, whereby it enacts HF-MSCs [14,28,74]. 

Macrophages play an essential role in the post-traumatic recruitment of HF-MSCs by arresting their enrollment into the injury postponing hair growth, although transplantation of dynamic macrophages is adequate for the enlistment of hair growth [28,75]. The job of Treg is also essential, wherein it introduces an abnormal state of Jag 1 from the Notch family, which influences the viable recovery of HF [76].

SVFs appear in a perfect cell populace for use in regenerative surgery due to the absence of immunogenic properties, their simplicity of acquisition, their multi-potential characteristic, the simplicity of separating them into different cell lines, and their significant potential for angiogenesis. SVFs have been created from wall cells situated in the perivascular portion, vascular smooth muscle cells, and pericytes—all associated with the arrangement of typical vasculature and are receptive to VEGF [77]. Normally, HFs encompassed by subcutaneous fat cells and by the dermis shape an inter-follicular dermal macro-environment, which is imperative for maintaining the best possible growth of bulge and follicle cells [24,59,78]. SVFs are vital for the activation of epidermal SCs, which they activate by discharging growth factors. The VEGF directs hair growth and the extent of the hair HF measure by stimulation of angiogenesis. HGF is associated with the span of the HC stages. The platelet-derived growth factor prompts and maintains the anagen stage, and IGF-I controls the hair growth cycle and hair cell separation [24,79]. Another heading for their activity is the stimulation of angiogenesis and enhancement of the blood supply to DPCs. Likewise, they have immunomodulatory and immunosuppressive properties via the collaboration among cells and the emission of prostaglandin E2 (PGE2), leukemia-inhibiting factor (LIF), and kynurenine [24,78]. 

The paracrine action of AD-SVFs is exceedingly complex, and the elements emitted by SCs have both a direct and an indirect impact on HFs. TB4 contributes to the activation of SCs in HF, improving their relocation into the follicle and separation. SDF-1 acts through an activation improvement of EGR-1, and it expands the cell tropism toward the follicle and builds angiogenesis. The activity of MCP-1, despite being an inflammatory factor, has a demonstrated tissue regenerative impact. A critical role of the microenvironment in the impact of paracrine factors in advancing the growth of the HF has also been underscored [78]. Huang et al. [80], in an investigation on rodents, found that an expansion of AD-SVFs to a culture of DPCs or core cells in the inner and external sheath improves their viability.

A huge increase in the regenerative potential was recorded in the investigation by Huang et al. [80], in which AD-SVFs were enhanced with LL-37, which is an antibacterial peptide normally present in wounds. Their review demonstrated a significant improvement in the nearby regenerative factors (i.e., the endothelial growth factor, thymosin beta-4, monocyte chemo-attractant protein-1, and stromal cell-inferred factor-1). A significant improvement in the growth of HFs, in both in vitro and in vivo creature models, was also observed [77,78,79,80,81]. 

Physiologically, fat tissue encompassing HFs assumes a critical role in extending the anagen stage. Adipocytes PCs have been found to increase during the progress from the telogen to the anagen stage around the HF [59,79]. Unfortunately, two-dimensional (2D) cultures of DPCs lose their hair formation capability in culture, which is why they require maintenance of their spheroidal forms (3D) [82,83]. This is a challenge to mature methods that mimic in vivo conditions, which both maintain the 3D structure of the cells and contain a special medium, which imitates a natural niche rich in growth factors [84].

The increase in the thickness of the subcutaneous layer during the advanced hair growth stage (anagen) was contrasted with the thickness in the resting stage (telogen). SVFs invigorate HFs through peroxisome proliferator-activated receptors, in which three isoforms have been found on their surfaces (PPARα, PPARγ, and PPARδ) [79]. Mature adipocytes negatively affect HF cell expansion and the multiplication of fibroblasts encompassing the HF in concurrent culture frameworks [24,85]. 

Strangely, a change in the adipocyte cell line properties can cause skin and hair issues. Lipid disorders can cause deficits in the skin structure and function. Overexpression of human apolipoprotein C1 (APOC1) with hyperlipidemia in transgenic mice causes hair growth issues corresponding to the grade of expression of human APOC1 genes in the skin [24,86]. 

Hypoxia, which is not lethal to MCs, improves the generation of growth factors for the ADSCs: VEGF, PDGF, HGF, and IGF-II [84,87]. The impact of hypoxia on AD-SVFs was analyzed in an investigation by Park et al. [87], in which SVFs were passaged multiple times with CO_2_ and subcutaneously injected into mice to observe induction of the anagen stage, and multiplication of human follicular cells of the DP and keratinocytes. Hypoxia produced a discharge increase in the insulin-like growth factor-restricting protein-1 and protein-2 (IGFBP), macrophage state invigorating element (M-CSF), M-CSF receptor, PDGF receptor-β, and VEGF, while the emission of the epidermal growth factor was lesser [87]. 

## 7. Clinical Intra-Surgical Application of PRP in HL and AGA

Many papers on PRP have been published, yet the outcomes are frequently conflicting. The authors take the perspective of not discussing PRP in general, though it is advisable to distinguish the different types of PRP relying on their cells content and fibrin design. Therefore, it is conceivable to recognize:

(1) Leukocyte-poor PRP (LP-PRP) or pure platelet-rich plasma (P-PRP): Suspension without leukocytes and with a low-density fibrin after induction;
(2) PRP and leukocyte (L-PRP): Suspensions with leukocytes and a low-density fibrin after induction (the largest of the commercial packages);
(3) Leukocyte-poor platelet-rich fibrin (LP-PRF) or pure platelet-rich fibrin (P-PRF): Suspension without leukocytes and a high-density fibrin;
(4) Leukocytes and platelet rich fibrin (L-PRF) or second-era PRP products are arrangements with leukocytes and a high fibrin density.

As discussed, there are too many protocols for the preparation of PRP depending on the different times for centrifugation and RPM used, the number of platelets, the accessibility of growth factors, and chemokines. Additionally, wide biological (among patients) and temporal (day-by-day) variation have been reported in the methods [88]. Thus, it is hard to evaluate which kind of PRP planning is better for clinical indications [89]. 

Diverse PRP products may be adequate for treating distinctive kinds of balding. The clinical efficacy of PRP is still under discussion, and a standardized protocol has not yet been created [90]. Doctors should choose the appropriate PRP preparations given their bio-molecular characteristics and clinical indications [91]. 

As of late, the use of low-level laser treatment (LLLT) has been proposed as a treatment for AGA and enhancing hair regrowth. The authors proposed LLLT 15 days after each treatment to stimulate hair regrowth during the HF-MSCs and PRP treatment, and every three weeks after the last treatment for a period of six months. Regarding this field, 11 papers were reviewed by Afifi et al. [92], where nine papers assessing hair count/hair density found noteworthy improvements in the men and women following LLLT treatment. Hair thickness and rigidity were found to be improved in two papers. Patient satisfaction was also accounted for in five of the works. 

Autologous platelet-rich plasma (A-PRP) is now connected with enhanced surgical results and lower repeat rates when used in the gingival retreat and keloid treatments, respectively [93,94]. In dermatological uses, differences were discovered when PRP treatments were performed with the activated autologous PRP (AA-PRP) instead of the non-activated A-PRP. At the point when A-PRP is used with autologous thrombin to yield AA-PRP, healing of bone exposure [95], chronic injuries as severe hidradenitis suppurativa [96] and shorter recuperation times were observed for profound burns [97,98]. Similarly, laser use for acne produces subjectively better outcomes with fewer reactions when performed in combination with either topical or intradermal use of calcium-activated PRP [99]. These outcomes might be ascribed to the discharge and concentrations of alpha-granule proteins, including growth factors and cytokines, that stimulate cell separation and proliferation, angiogenesis, and vascular modeling [100]. 

In the treatment of HL, the topical use of AA-PRP with the collected follicles preceding implantation has been shown to build their survival rate by 15% [101]. Patients treated with calcium gluconate-initiated PRP displayed expanded hair thickness three months post-medical procedure, with terminal hair thickness (measurement > 40 μm) increasing by 19% during that time [102]. These discoveries were affirmed in an examination of AGA patients treated with calcium-activated PRP over a span of one year [103]. After 12 weeks from the last infusion of PRP, hair thickness reached a 19% expansion over the baseline estimations. At the one-year point, hair thickness decreased to 7% above the standard estimation, although this was still established as a significant increase in hair thickness in contrast to the baseline esteems [103]. 

The growth factors (GFs) acquired by the degranulation of the alpha-granules appear to stimulate hair regrowth. In detail, IGF-1 stimulates the multiplication of cycling Ki67+ basal keratinocytes [104,105], whereas TGF-β1 secures the proliferative capability of basal keratinocytes by repressing cell growth and terminal separation [106,107]. PDGF-AA increases the hair inductive action of DPCs when used in combination with fibroblast growth factor 2 (FGF-2) [108,109]. VEGF stimulates angiogenesis, and PDGF-BB is a strong chemo-attractant for wound macrophages and fibroblasts by stimulating these cells to discharge endogenous growth factors, including TGF-β1, which advances new collagen synthesis [110]. 

DPCs harvested from the human scalp have shown improved proliferation, improved Bcl-2 and FGF-7 levels, activated ERK and Akt proteins, and up-regulation of β-catenin when cultured in an initiated PRP-enhanced growth medium [111]. Each of these elements decidedly impacts hair growth through cell multiplication to prolong the anagen stage (FGF-7) [29], stimulating cell growth (ERK enactment) [112], invigorating HF development (β-catenin) [113], and stifling apoptotic signals (Bcl-2 discharge and Akt actuation) [114,115]. The human scalp affected by AGA treated with PRP injections should show significant increases in cell activity. Histological examinations of A-PRP- and AA-PRP-treated scalps from our past work [116] provide such clinical proof. In both patient populaces, the authors observed an enhancement in the number of follicular bulge cells and follicles, epidermal thickening, enhanced vascularization, and a higher number of Ki67+ basal keratinocytes in the PRP-treated scalp tissue compared to the placebo. 

Hair regrowth in a clinical evaluation demonstrated a positive reaction to treatment with A-PRP in patients showing significative improvements in hair density and hair count in the treated zone over the control zone (treated with the placebo). Differences between the 12-week follow-up hair counts and the baseline hair counts were observed. These hair growth parameters were higher in the A-PRP treatment group than in the AA-PRP treatment populace, as reported in past preliminary data reported by Gentile et al. [116]. Specifically, three-month hair density estimations for patients treated with A-PRP and AA-PRP were 65 ± 5 and 28 ± 4 hairs/cm^2^, respectively. The outcomes established a 31 ± 2% improvement in hair density when the A-PRP treatment was performed versus a 19 ± 3% improvement in hair density when the AA-PRP treatment was performed, with a significant difference in hair growth (*p* = 0.0029). The increase in the hair growth parameters for A-PRP over AA-PRP may mirror the proficiency of in vivo thrombin in activating platelets and the body to distribute the contents of the activated platelets compared to in vitro calcium activation and infusion. The delivery of A-PRP may empower the production of thromboxane A2 (TXA2) by the platelets once they are activated in vivo, which would activate additional platelets and amplify platelet aggregation [117].

## 8. Clinical Intra-Surgical Application of HFSCs in Hair Loss and Androgenic Alopecia

It is hard to find particular strategies to enhance the regeneration of HF under conditions suitable for an adult individual. Given the knowledge on ECs and dermal cells, and their relationship in the midst of embryonic hair age and adult hair cycling, various scientists have tried to obtain mature hair follicles using techniques and procedures that rely on the causes for AGA [42,118]. In a preliminary examination [53], the authors developed another procedure to separate HFSCs using minimal manipulation, depending on the centrifugation of pieces of human hair follicles without cell expansion or enzymatic digestion. They reported the tallying of these cells and the preliminary results obtained from injections of micrografts containing HFSCs in the scalps of patients affected by AGA showed improvements in hair density. Gentile et al. [53] reported the amount of CD44+ cells from DP and the level of CD200+ cells from the bulge obtained using the customized centrifugation of 11 punch tests [53]. They also reported the microscopic evaluation of punch biopsy samples, determined by cytospin and immunocytochemistry, histological examination using hematoxylin and eosin staining, and clinical appraisal. The authors now seek to discuss improvements to the current systems available for the recovery and regeneration of hair follicles, focusing on systems permitting neo-genesis of hair follicles in adult individuals by using isolated cells and biotechnologies [53]. 

Examinations were performed using rodent cells, particularly of embryonic or infant origin. No fruitful procedure to produce human hair follicles from adult cells has been found. Possibly, the most crucial point is creating 3D culture conditions reflecting the structure of living tissue. It is necessary to improve the culture conditions that allow the expansion of specific cells while preserving their inductive properties, as well as procedures for picking masses of epithelial stem cells (ESCs), which should provide the principal instruments to overcome the difficulties constraining human HF neo-genesis [42]. These cells give the impression of being arranged in the bulge district of human hair follicles.

### Hair Follicles and HF-MSCs Regenerative Mechanisms in Hair Loss and Androgenic Alopecia

HFs are known to have a well-characterized niche for grown-up SCs—the bulge, which contains ESCs and melanocytic SCs [119]. SCs in the hair bulge, an obviously-differentiated compartment inside the lower portion of hair follicles, can produce inter-follicular epidermis, HF structures, and sebaceous glands [120,121]. The bulge ESCs can also reconstitute in a simulated in vivo framework to a new HF [122,123].

Yu et al. [119] showed that follicles of human hair contain a SC populace that can be identified in the smooth muscle cell, as well as neuron and melanocyte heredities in the induction medium. Their analysis demonstrated that Oct4+ cells are present in human skin, and the greater proportion are positioned in the HFs in vivo. Oct4 has a place in the family of POU-domain transcription factors that are regularly communicated in the pluripotent cells of the developing embryo and that mediate pluripotency [124]. Additionally, human HFs contain multi-potent SCs other than ESCs and melanocytic SCs, and these cells are positioned in the bulge region. These cells indicate promising plasticity in ex vivo and in vitro conditions, making them potential candidates for cell engineering and cell substitution treatments [124]. 

Each mature HF is a regenerating framework that physiologically experiences cycles of growth (anagen), relapse (catagen), and rest (telogen) at various times in an adult’s life [125]. In catagen, HFSCs are maintained in the bulge. At that point, the resting follicle re-enters anagen (regeneration) when legitimate molecular signals are provided. During late telogen to early anagen change, signals from the DP stimulate the hair germ and quiescent bulge SCs to activate [29]. Numerous paracrine components are involved in this crosstalk at various HC stages, and some signaling pathways are involved [126,127,128]. In anagen, SCs in the bulge produce an ascent in hair germs, and at that point, the transient increasing cells in the grid of the new follicle proliferate rapidly to frame another hair filament [129]. The authors felt the need to better understand in which stage it is necessary to act.

Regeneration of HFs was observed in humans [130] when dermal sheath tissue was used, which was adequate to regenerate the DP structure. After implantation, the whisker DP was equipped to promote HF regeneration as holding the data to decide hair fiber type and follicle size [131]. In an examination [42], the authors prepared a dermal–epidermal skin substitute in a research facility by seeding an acellular dermal grid with cultured HF-ESCs and DPCs, both obtained from an adult human scalp. These constructs were grafted onto a full-thickness wound produced on bare mice skin. In 14 days, histological structures reminiscent of a wide range of phases of embryonic HF improvement were seen in the grafted region. These structures demonstrated concentric cellular layers of human origin and expressed k6hf, a keratin present in the epithelial cells of the companion layer. Despite completely mature hair follicles not being observed, these outcomes demonstrated that both epithelial- and dermal-cultured cells from the adult human scalp in a dermal scaffold could create in vivo structures that reiterate embryonic hair improvement.

Kalabusheva et al. [132] combined postnatal human DPCs and skin epidermal keratinocytes (KCs) in a hanging drop culture to mature a recreated HF germ. The procedure relied on DP cell hair-affecting properties and KC self-affiliation. The authors examined two protocols of aggregate gathering. Blended HF germ-like structures demonstrated the initiation of epithelial–mesenchymal cooperation, including Wnt pathway establishment and expression of follicular markers. They analyzed the effect of conceivable DP cell parts, including dissolvable segments and extracellular matrix (ECM) molecules during the time spent on the organoid collection and growth. Their results demonstrated that dissolvable parts had limited impact on HF germ age and Ki67+ cell scores inside the organoids, despite BMP6 and VD3 viably maintaining the DP character in the monolayer culture. Aggrecan, biglycan, fibronectin, and hyaluronic acid (HA) altogether improved cell multiplication in the DP cell monolayer culture with no effect on the DP cell character. A substantial part of ECM compounds confined the growth of cell aggregates, while HA propelled the formation of greater organoids. 

Talavera-Adame et al. [133] uncovered the bio-molecular pathway involved in cell treatment. In particular, they exhibited that Wnt/β-catenin signaling was central to the growth and upkeep of DPCs [134,135]. The augmentation of Wnt signaling in DPCs is an essential factor that upgrades hair regrowth [134]. Specifically, in Pirastu et al. [136], androgen receptor flagging was involved in seven genes at six loci. Three primary gatherings were discovered: genes connected to Wnt flagging (*RSPO2*, *LGR4*, *WNT10A*, *WNT3*, *DKK2*, *SOX13*, *TWIST2*, *TWIST1*; *IQGAP1*, and *PRKD1*), genes involved in apoptosis (*DFFA*, *BCL2*, *IRF4*, *TOP1*, and *MAPT*), and heterogeneous gathering, including the androgen’s receptor and TGF-beta pathways (*RUNX3*, *RUNX2*, *ALPL*, *PTHLH*, *RUNX1*, *AR*, *SRD5A2*, *PDGFA*, *PAX3*, and *FGF5*). Although a wide range of pathways have been implicated in the advancement of AGA, their outcomes suggest that, notwithstanding the androgen receptor pathway for which they affirm a fundamental function, the Wnt and apoptosis pathways assume a major role. AGA is described by shorter growth (anagen), which has been related to the increased apoptosis of the HFs. This outcome suggests that the anagen stage shortens as a result of contrasts in the genes managing the apoptosis. The Wnt pathway is involved in the advancement of telogen (resting) to the anagen (growth), and in the fate of the SCs in the hair bulge, which are both dysregulated in balding tissue. Finally, hair loss chance loci in Wnt ligand biogenesis and trafficking and class B/2 (secretin family receptors) pathways were additionally connected with height, despite none of the individual loci in these pathways being crucial, suggesting a far-reaching impact. Along these lines, hairlessness indicates pathway-explicit hereditary relationships that provide a potential natural premise to the observed epidemiological connections. Pathway-specific hereditary relationships can help us to disentangle the mutually-organic pathways supporting complex pathologies [136].

## 9. Studies on Using Stem Cells from Wharton’s Jelly 

What are the advantages of using SCs from Wharton’s jelly compared to other MCs? Wharton’s jelly is a particular source of SCs due to its accessibility, vast pool of donors, non-invasive and easy harvesting, no hazard to the donor, no moral impediments, limited immunogenic potential, and high multi-potential separation ability [137,138]. In addition, the exposure to infectious agents seldom happens, which ensures security to the contributor [139]. The decellularized Wharton’s jam matrix (DWJM) (fresh jelly exposed to two cycles of osmotic shock, alternately with a hypertonic suspension of NaCl, mannitol, MgCl_2_, and KCl with an osmolarity of approx. 1.275 mOsm/L, centrifuged at 5000 rpm at 4 °C with a hypotonic arrangement of 0.005% Triton X-100) can provide a characteristic scaffold to SCs as a biocompatible matrix, which bolsters their viability, triggering conglomeration of MCs. DWJM contains TGF-β, collagen I, fibronectin, and tenascin, which might be in charge of condensing the added WJMSC in a few regions of the DWJM. In synthesis, DWJM is a natural biocompatible 3D matrix that guarantees adhesion, penetration, growth, and multiplication of cells, both in vitro and in vivo. To summarize, the report presented DWJM as a normal 3D scaffold that can be used in tissue designing and as a regenerative drug [140].

## 10. Concluding Remarks

Maintaining a pool of SCs is vital for tissue homeostasis and harm fix. Their divisions are not frequent in mature organisms, and their greater part are in a lethargic state. As such, it is vital to comprehend the components of their activation and induction, which will allow for the use of multi-potent cells in regenerative plastic surgery and hair regrowth. Their use is complicated by the fact that the expression of receptors on the various growth factors and the effect of the microenvironment may vary.

Not all target points in SC therapy have been distinguished. The information provided in this review features the useful impacts of AD-SVFs and PRP on hair regrowth, notwithstanding the preliminary positive outcomes from HF-MSCs. The activation and increase of Wnt signaling in DPCs is the crucial factor that enhances renewed hair growth. 

The current knowledge in biology, the limits of past translational research that included development of pre-clinical and clinical models, harnessing of new strategies for more accurate imaging, and biomarker-based diagnostics will provide a strong basis to advance viable clinical approaches for regenerative aims in hair tissue engineering. Moreover, larger, randomized, double-blinded, controlled trials are needed to optimize cell administration protocols and to confirm the early observations of promising clinical outcomes.
